# Reminders of the Past

**Published:** 1892-11

**Authors:** 


### Reminders of the Past.

One of the most interesting exhibitions in connection with the recent Orientalist Congress in London is a collection of tools used by workmen in building the pyramids of Egypt. They were gathered and are exhibited by the illustrious Egyptologist, Mr. Flinders Petrie. These utensils indicate that ancient workmen had an astonishing acquaintance with many tools which we have been accustomed to consider essentially modern. Among the exhibits are solid and tubular corundum-tipped drills and straight and circular saws and chisels described as "not a bit inferior to those now used."

Black glass was once used for mirrors as well as transparent glass with some black substance on the back. It is related that the Spaniards found mirrors of polished black stone, both convex and concave, among the natives of South America.

A company has been formed at Christiania, Norway, to reproduce an exact model of the old Viking boat that was discovered some years ago in an ice floe.

Brandy is an invention of the French and has been known to the world for nearly 600 years.

### Patent Medicines in Turkey.

The Turkish Government, as is well known, has prohibited the entrance of remedies whose composition is unknown. The merchants who deal in these goods recently petitioned the government to abrogate this law but their request was not granted. —*Medical Review.*

### Ratio of Physicians to Population.

Dr. P. H. Millard, of Minnesota, (*Med. Review*), in a paper recently read before the American Academy of Medicine, gave the following as the ratio of physicians to population in various parts of the world: Sweden, 1 to 7,000; Italy, 1 to 3,500; Germany, 1 to 3,000; Austria-Hungary, 1 to 2,400; France, 1 to 2,000; United States, 1 to 600.

